# Parkinsonism secondary to metastatic lesions within the central nervous system: a case report

**DOI:** 10.1186/1752-1947-4-218

**Published:** 2010-07-21

**Authors:** Eduardo Hortelano, Christian Perea, Esther Uña, Amelia Cebayos, Patricia Diezhandino, Montserrat González

**Affiliations:** 1 Radiotherapy Service, Clinical University Hospital, C/Ramon y Cajal n° 3, 47005 Valladolid, Spain; 2 Medical Oncology Service, Clinical University Hospital, C/Ramon y Cajal n° 3, 47005 Valladolid, Spain; 3 Neurology Service, Central University Hospital, C/Celestino Villamil s/n, 33006 Oviedo, Spain

## Abstract

**Introduction:**

Colorectal cancer is one of the most common human diseases worldwide, and metastases are detected in approximately 20% of patients at diagnosis. Brain metastases occur in only 4% of cases, however, and usually present with hemiparesis or other motor or sensory symptoms. There have been only a few reports of parkinsonism secondary to a brain tumor-related mass effect.

**Case presentation:**

We present an unusual case of parkinsonism secondary to multiple brain metastases. A 57-year-old Caucasian man had recently been diagnosed with primary carcinoma of the colon and had multiple metastases in the lungs and liver. He subsequently developed bilateral symmetrical parkinsonism, and multiple brain tumors were detected by computed tomography scanning. The condition of our patient deteriorated rapidly, and he became akinetic and dependent for all activities of daily living. He was followed up and treated at home by our palliative care unit team and died two weeks after the onset of his neurologic symptoms.

**Conclusion:**

Although primary and secondary brain tumors are uncommon causes of parkinsonism, their clinical presentation may resemble that of idiopathic Parkinson's disease. An awareness of this rare differential diagnosis is therefore important in ensuring early diagnosis and treatment, thus improving prognosis and quality of life. A rapid progression in neurologic symptoms was observed in our patient, and clinicians should be alert to this atypical presentation of secondary parkinsonism.

## Introduction

Colorectal cancer is one of the most common malignant diseases worldwide, and approximately 20% of patients have metastases at diagnosis [1]. The liver, lung and peritoneum are the most common sites of metastatic spread. Brain metastases occur in only 4% of cases, however, and are usually present with hemiparesis or other motor or sensory symptoms.

There have been very few reports of parkinsonism secondary to a brain tumor [2] or other brain lesion with a mass effect [3]. Supratentorial lesions involving the caudate-putamen or the striatonigral tract are the most common lesions described in these reports, but occasionally no lesion is identified.

It is assumed that mass effect plays an important role in the pathological effects of brain lesions.

## Case presentation

A 57-year-old Caucasian man born in Spain was admitted to hospital with a two-month history of asthenia and diffuse abdominal pain that was most intense over the left flank. His medical history was unremarkable, and there was no family history of cancer or Parkinson's disease. Abdominal distension was observed on clinical examination. Blood samples were taken to measure the full blood cell count and general biochemical parameters. Moderate anemia was detected (hemoglobin 8 g/dL). Further tests, including colonoscopy, revealed a bleeding polypoid mass that was partially obstructing the lumen of the sigmoid colon. A biopsy confirmed non-mucinous adenocarcinoma.

A computed tomography (CT) whole-body scan detected multiple nodules in both lungs and in the liver, as well as the presence of multiple enlarged lymph nodes in all visible compartments.

Our patient was diagnosed with stage IV (TNM [tumor, node, metastases] of the American Joint Committee on Cancer sixth edition classification) advanced adenocarcinoma of the colon. He received a blood transfusion, and the surgical team performed a palliative ileocolic bypass with primary anastomosis.

Our patient's clinical signs and symptoms improved over the course of the pre-operative period, and he was discharged home one week after surgery. Four days after discharge, our patient developed pyrexia and testicular pain and tenderness. He was readmitted to hospital with suspected orchitis, and intravenous (IV) broad-spectrum antibiotic therapy was commenced. Spontaneous drainage of a scrotal abscess relieved our patient's symptoms, and he was discharged from the hospital.

During the last three days of this second admission, our patient's wife had noticed that he had begun to walk with small steps, was slower in his movements, had become clumsy and that his posture was generally quite rigid. These neurologic signs worsened post-discharge, and our patient developed bilateral bradykinesia, postural instability and a wide-based gait with freezing. By this stage, he required assistance with the activities of daily living (ADLs). General physical and neurologic examination revealed a very debilitated patient. His facial expression was mask-like and impassive with slow eye movements, and he was dysarthric. A resting tremor of both hands was observed. He walked with extreme difficulty and required the assistance of two people.

His pace was very slow, and he took small, wide-based steps. He had a festinating gait with an absence of bilateral arm swinging. No obvious pulsion was observed, but he adopted a bilaterally dystonic posture. Generalized hyper-reflexia and plastic rigidity with bilateral cogwheeling were demonstrated, but no cranial nerve palsies were detected. No severe cognitive deficits could be elicited, although his responses were delayed, and he appeared disinterested.

**Figure 1:**
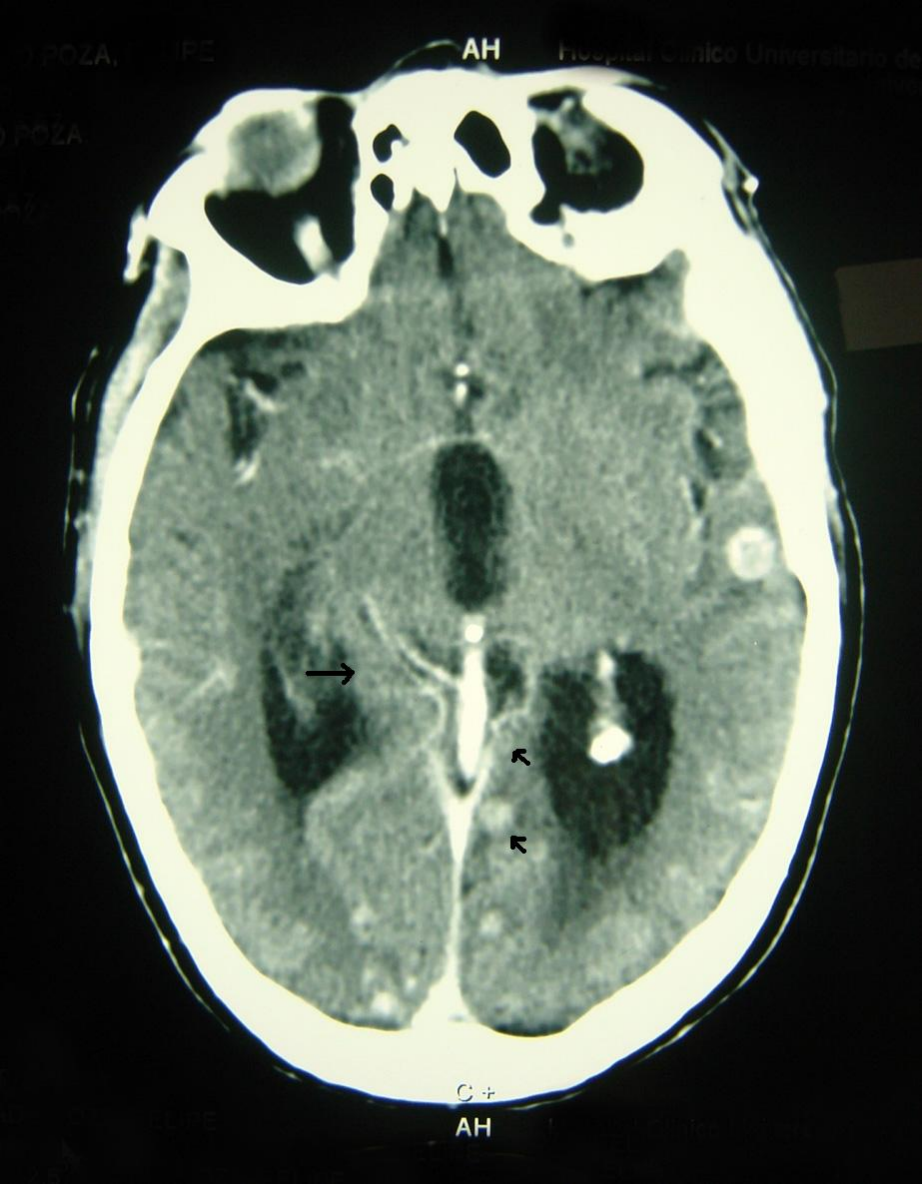
**Multiple brain metastases secondary to adenocarcinoma of the colon affecting several hemispheric areas**.

**Figure 2:**
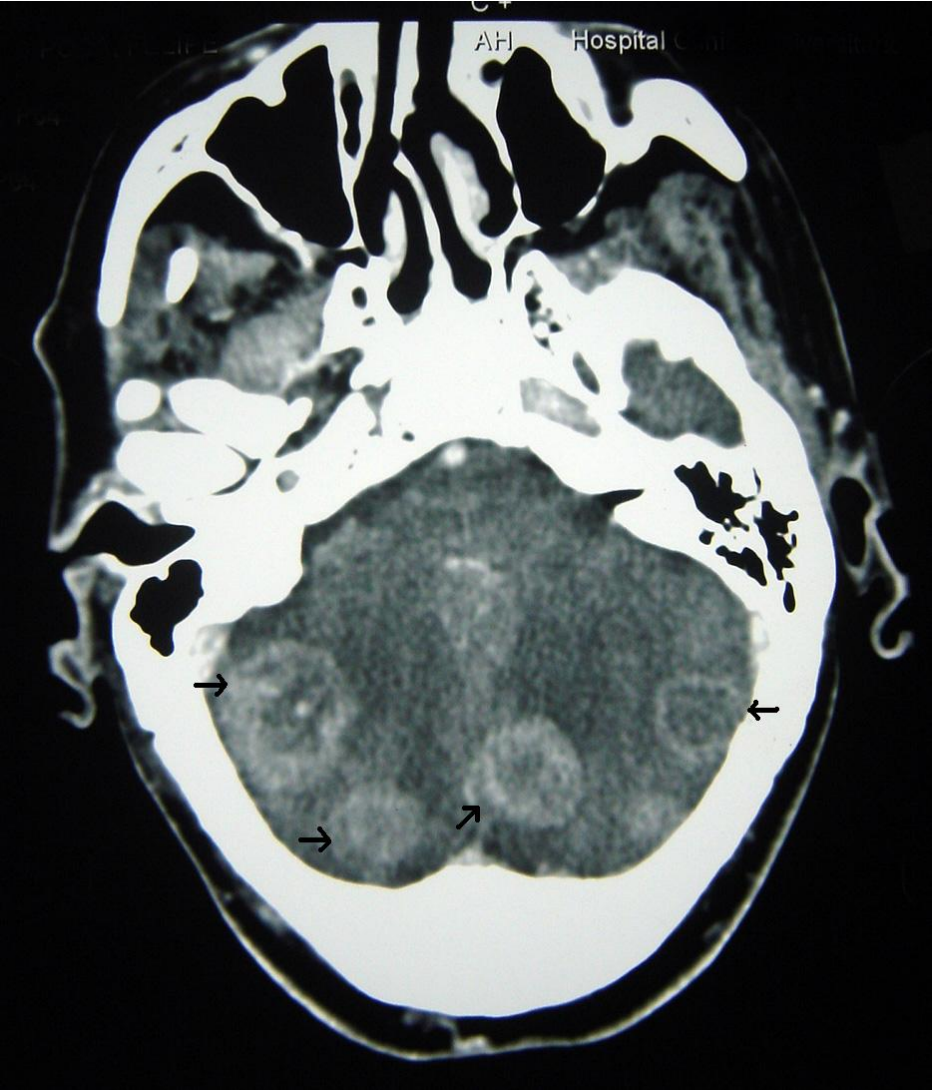
**Multiple metastases of colon cancer affecting other brain regions**.

All vital signs were unchanged. His family reported the occurrence of nighttime urinary incontinence and that he was now completely dependent regarding all ADLs. Because our patient had no history of neurologic disease or hypertension, brain metastases were suspected, and treatment with high-dose IV steroids started. This resulted in an obvious improvement in the neurologic signs. Our patient's thyroid-stimulating hormone level was found to be normal. Emergency CT of the brain revealed the presence of multiple brain lesions with contrast enhancement and mass effect, which were surrounded by dense vasogenic edema. These features are known to be suggestive of metastases (Figures 1 and 2). It was not possible to perform magnetic resonance imaging of the brain because of our patient's resting tremor. After a brief period of improvement in response to steroid therapy, his condition deteriorated rapidly, and it was not possible to begin radiotherapy or a trial of L-DOPA (a dopamine agonist). The rapidity of our patient's decline also precluded examination of a serum paraneoplastic autoantibody panel.

## Discussion

Central nervous system metastases occur more commonly in rectal cancers than in colonic cancers [4]. The development of brain metastases usually occurs as a late event in patients with multiple distant metastases [5], as reported by Hammoud *et al. *[3] in a retrospective study of 100 patients with brain metastases secondary to colorectal adenocarcinoma. Although a median interval of 26 months between the diagnosis of the primary cancer and diagnosis of brain metastases was found in the study by Hammoud *et al. *[3], the corresponding interval in our patient was very short. Our patient presented initially with advanced extra-neural disease without neurologic symptoms. It is probable that the shortness of the interval between diagnosis of the primary tumor and the appearance of neurologic symptoms was attributable to physical and psychological stressors secondary to the high disease burden and post-operative complications; these factors may have decreased our patient's threshold of neurologic resistance. It is striking that neurologic symptoms were the only clinical manifestations of metastatic disease in this patient.

Reports of brain metastases in patients with colorectal cancer are rare, and the typical clinical presentation described for these patients has been the development of motor or sensory deficits. There have been very few reports of parkinsonism secondary to a brainstem mass. The first report of parkinsonism secondary to brain lesions was published by Blocq and Marinesco in 1893 [6]. This report described a patient with tuberculosis of the brain. Since then, approximately 90 cases of tumor-induced parkinsonism have been reported. Most of these reports have described supratentorial tumors that directly involved or indirectly affected the basal ganglia or the nigrostrial tract. The most frequent causes of neoplastic parkinsonism are astrocytomas, meningiomas, craniopharyngiomas, colloid cysts and (less frequently) metastases [7]. Several cases of parkinsonism secondary to primary brain lymphoma have been described. In one of these cases, no brain lesion was detected during CT scanning, but a diffuse B-cell lymphoma with selective infiltration and neuronal destruction of the substantia nigra was found post-mortem [8,9]. Seven other cases involved a midbrain mass that presented with hemibody hyper-reflexia, transient diplopia and unilateral parkinsonism. Other studies have described similar features. Many of the patients described in these studies have been reported to be responsive to L-DOPA [8].

Pramstaller *et al. *[8] hypothesized that the existence of comorbid Parkinson's disease or a mass effect involving the contralateral substantia nigra may explain bilateral involvement. In our case of disseminated brain metastasis, although not visualized in the basal ganglia via CT scanning, it was believed that microscopic infiltration with secondary compressive effects occurred. The fact that this was not detected during CT scanning may have been attributable to the density of the surrounding edema. Our rationale for this hypothesis is the fact that our patient's symptoms responded to high-dose steroids and that his medical history was unremarkable. The early recognition of parkinsonism that is secondary to an intra-cranial tumor has important implications for clinical management. CT scanning of the brain therefore plays an important role in the differential diagnosis of patients with parkinsonian symptoms. Although movement disorders secondary to paraneoplastic syndromes have been described [10], we were unable to investigate this differential diagnosis in our patient because of the rapidity of his clinical decline. However, we consider it highly unlikely that paraneoplastic syndromes were implicated in the present case because such syndromes are extremely rare, and the presence of multiple brain lesions was clearly demonstrated.

Our patient had multiple brain metastases involving the bilateral cerebral hemispheres and the cerebellum. Although we did not detect any lesions involving the basal ganglia on CT, it is highly unlikely that our patient had an early phase of idiopathic Parkinson's disease. The rapid progression of neurologic symptoms and subsequent advanced colonic adenocarcinoma are both suggestive of neoplastic parkinsonism. Long-term follow up is important in differentiating between neoplastic parkinsonism and idiopathic Parkinson's disease in cancer patients.

## Conclusions

Brain tumors are an uncommon cause of parkinsonism. However, the presence of an advanced carcinoma with rapid progression of parkinsonian symptoms should lead one to suspect neoplastic parkinsonism.

Because the clinical presentation of tumoral parkinsonism may resemble that of idiopathic Parkinson's disease, an awareness of this rare differential diagnosis is important in ensuring early diagnosis and treatment, thus improving the prognosis and quality of life of these patients.

## Consent

Written informed consent was obtained from the patient's next of kin for publication of this case report and any accompanying images. A copy of the written consent is available for review by the Editor-in-Chief of this journal.

## Competing interests

The authors declare that they have no competing interests.

## Authors' contributions

EH, CP, AC, PD and MG were involved in the acquisition of data and in drafting the manuscript.

EU was involved in the conception and the drafting of the manuscript and has reviewed its scientific content. EU has approved submission of the final version of the manuscript. All authors read and approved the final version of the manuscript.

## Author information

**EH**, trainee radiation oncologist, University Hospital of Valladolid.

**CP**, trainee radiation oncologist, University Hospital of Valladolid.

**EU**, medical oncologist, University Hospital of Valladolid and Professor of Oncology, Valladolid School of Medicine.

**AC**, trainee radiation oncologist, University Hospital of Valladolid.

**PD**, radiation oncologist, University Hospital of Valladolid.

**MG**, neurologist, Central University Hospital of Oviedo.
